# Using a Teaching Intervention and Calibrated Peer Review™ Diagnostics to Improve Visual Communication Skills

**DOI:** 10.1007/s10439-017-1946-x

**Published:** 2017-11-03

**Authors:** Ann Saterbak, Anoosha Moturu, Tracy Volz

**Affiliations:** 1 Department of Biomedical Engineering, Duke University, Durham, NC USA; 2 Baylor College of Medicine, Houston, TX USA; 3 Program in Writing and Communication, Rice University, 6100 Main St – MS 630, Houston, TX 77005 USA

**Keywords:** Tissue culture, Data analysis, Data interpretation, Peer review, Assessment, Graphs

## Abstract

Rice University’s bioengineering department incorporates written, oral, and visual communication instruction into its undergraduate curriculum to aid student learning and to prepare students to communicate their knowledge and discoveries precisely and persuasively. In a tissue culture lab course, we used a self- and peer-review tool called Calibrated Peer Review™ (CPR) to diagnose student learning gaps in visual communication skills on a poster assignment. We then designed an active learning intervention that required students to practice the visual communication skills that needed improvement and used CPR to measure the changes. After the intervention, we observed that students performed significantly better in their ability to develop high quality graphs and tables that represent experimental data. Based on these outcomes, we conclude that guided task practice, collaborative learning, and calibrated peer review can be used to improve engineering students’ visual communication skills.

## Introduction

### Background on Visual Communication in Engineering

#### Importance of Visual Communication in Engineering

In *The Engineer of 2020: Visions of Engineering in the New Century* the National Academy of Engineering identifies communication skills as a top priority for engineering graduates to be successful.^18^ In addition to written and oral skills, engineers need to possess visual communication competencies such as sketching, modeling, rendering, and data presentation.^4^,^9^ To develop these competencies, many engineering faculty assign reports, presentations, and posters that feature images, computer-aided design drawings, figures, and tables, yet it is not clear students receive much instruction on how to create effective visuals.

Some engineering faculty have found that students are ill-prepared to visualize and interpret data and have taken the initiative to explicitly teach students at every level how to become more proficient in these areas.^6^ Taylor and Pulford, for example, note that prior to enrolling in capstone design, engineering students receive little instruction in design principles or core skills such as observation and envisioning, which aid in the preparation and interpretation of visuals.^25^ Other engineering faculty have attempted to address this problem earlier in the engineering curriculum. Sherwood and Atabile were dissatisfied with the quality of the figures and tables created by juniors in a mechanical engineering design course and implemented a rubric to signal their expectations and to evaluate students’ work.^24^ Dupen introduces first-year students to data presentation in lab reports through a combination of mini-lecture, assignment-specific instructions, and repeated practice.^8^ A scaffolded approach to learning that involves frequent practice and feedback is needed to achieve a high level of visual communication competency.

#### Prior Work and Intervention to Target Course Learning Outcomes

Recognizing the importance of multi-modal communication, bioengineering faculty at Rice University partnered with professional communications faculty to develop and implement a vertically integrated, communication-enhanced curriculum.^21^,^23^ As part of this curriculum, the authors created an assignment in BIOE 342, a tissue culture laboratory course, in which students develop technical posters summarizing their experimental results.^26^ This assignment is specifically designed to develop students’ skills in data presentation and analysis of results. We integrated a tool called Calibrated Peer Review™ (CPR) with the poster assignment, which allowed us to capture students’ evaluations of the poster drafts. The CPR data combined with instructor evaluations made it possible to conduct a fine-grained analysis of the posters’ strengths and weaknesses.

In 2007 we launched a research project to investigate the level of agreement—or lack thereof—between the instructor’s evaluations of students’ posters and students’ evaluations of their own and their peers’ posters.^26^ Data collected *via* CPR between 2007 and 2009 indicated that students were struggling to construct technical arguments and to create effective visual displays of their results. The CPR data confirmed our perception that students were skilled at evaluating their peers’ posters on lower-order cognitive tasks such as the formatting of figures and tables and simple reporting, but they performed poorly on higher-order cognitive tasks such as analysis, synthesis, and evaluation. More specifically, students struggled with tasks such as condensing and presenting graphical data, correctly interpreting graphical representations of data, and synthesizing and stating key results, especially as they relate to linking experimental variables with measured cellular effects.^22^,^26^

Therefore, in 2010, we initiated a pedagogical intervention to target two learning outcomes (LOs): (1) Develop high quality graphs and tables that represent experimental data, and (2) Correctly interpret and clearly summarize the experimental results. This paper describes CPR, the tissue culture laboratory, and the classroom activities directed at the two LOs. The paper also discusses the results of the intervention on student performance.

### Background on Calibrated Peer Review™ (CPR)

CPR facilitates asynchronous, anonymous electronic peer review.^26^ It was designed to facilitate peer-graded writing assignments in large lecture courses and has been widely adopted by educators in a variety of disciplines including biology, chemistry, engineering, and medical education.^1^,^12^,^14^,^15^ Faculty have used CPR to assess essay writing,^28^ lab reports and design reports,^12^ technical writing,^16^ and course content.^17^ We were early pioneers in the use of CPR to teach technical posters.^22^,^26^ Recent improvements to CPR 6 include a new interface specifically designed to support peer review of graphics and multi-media files.^5^,^6^,^27^

To complete the CPR assignment, students upload their poster drafts in the *Text Entry* stage. In the *Calibration* stage, students apply evaluation criteria to three sample posters, which have been selected and rated by the instructor. Students’ ratings of each sample poster must fall within a prescribed standard deviation established by the instructor before students can enter the *Review* stage. During the *Review* stage, students use the same criteria to evaluate three peers’ texts. After reviewing their peers’ texts, students complete the *Self*-*Assessment* stage. When the assignment concludes, students can view their results.

Instead of using CPR to replace instructor grading with peer grading, we use CPR in BIOE 342 to give students more experience with peer review and as a mechanism for formative assessment. Students receive credit for their critiques in CPR, but their poster grades are determined by the instructor.

### Educational Framework

#### Tissue Culture Laboratory

The Tissue Culture Laboratory (BIOE 342) is one of the required laboratory courses in the bioengineering curriculum that includes a significant communication component. The instructor established six course goals: (1) Demonstrate proficiency with tissue culture techniques, (2) Keep a laboratory notebook, (3) Perform assays to characterize cell viability and function, (4) Articulate links between changes in cellular environmental conditions and cellular function, (5) Improve communication skills, and (6) Refine critiquing skills. To achieve the first four goals, students learned sterile technique and performed viability, attachment, and proliferation assays using a fibroblast cell line. The experiments produced quantitative data such as cell number, cell density, or fractions. To meet the final two goals, students prepared a poster of their laboratory results and subsequently critiqued their peers’ posters. This 5-week, junior-level course carried 1 h of credit and enrolled approximately 13 students per section, with four sections offered per year. Students spent 6–10 h in lab each week and attended six 50-min lectures.

#### Standard Poster Assignment

This section describes the standard poster assignment prior to the intervention. In the context of this course, “poster” refers to 10–12 slides that present experimental objectives, methods, results, and conclusions. This format has two advantages over conventional conference-style posters. First, it focuses students’ attention on constructing a technical argument without having to tackle macro-level layout and design issues, which are addressed in other bioengineering courses. Second, it allows students to avoid printing costs. Students in the course do not present their posters orally at a poster session.

To prepare students, the bioengineering instructor introduced the assignment and expectations. Then, the communications instructor lectured on the poster genre, technical argument, and data presentation. Following this lecture, students met in small groups for 5–10 min to critique three poorly designed slides produced by former BIOE 342 students and reported their observations to the class. During standard instruction (2007–2009), these activities occurred in lecture period 5 (Table 1).

**Table 1:**
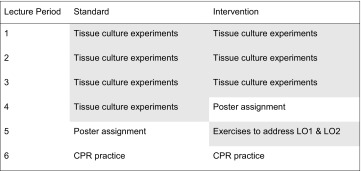
Comparison of course schedule before and after the intervention.

The intervention schedule removed one lecture period on tissue culture experimental details. In its place, an active learning lesson intended to increase proficiencies in LO1 and LO2 was added.

Later, students submitted poster drafts, which were graded by the instructor and evaluated by their peers using CPR. During the *Calibration* stage, students reviewed three sample posters based on a journal article that had similar tissue culture experiments to the ones the students had completed.^13^

To critique the posters in the *Calibration* and *Review* stages, students used 14 evaluation statements related to presenting data in figures and tables, interpreting data in words, and applying disciplinary conventions (Table 2). Based on the feedback students received from the instructor and their peers, they revised their posters for a final grade. The course grade was weighted heavily toward this assignment: poster draft (10%), CPR performance (10%), and revised poster (20%).

**Table 2:**
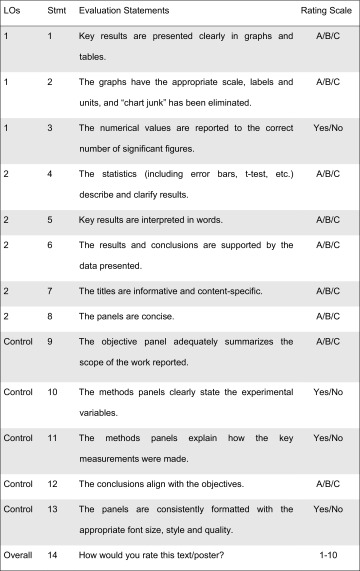
Evaluation statements.

Students and instructor used the statement number (Stmt), evaluation statement, and rating scale to critique the posters. A/B/C scale: *A* high/strongly agree, *B* moderate/neutral, *C* low/strongly disagree. Yes/No scale: *Yes* agree, *No* disagree. 1–10 scale: 1 is low, 10 is high.

#### Intervention Poster Assignment

To address specific areas of students’ underperformance in the technical poster assignment identified earlier,^24^ we implemented a pedagogical intervention in spring 2010. This type of intervention is based on task-based practice and cooperative learning and has been shown to improve student performance in a laboratory setting.^2^,^20^ The course outline, experiments, assignments, and relative weighting of the assignments remained the same during the intervention period (2010–2012).

The same 14 evaluation statements were used during the intervention period (Table 2). Statements 1–3 were linked to LO1: *Develop high quality graphs and tables that represent experimental data*, and Statements 4–8 referred to LO2: *Correctly interpret and clearly summarize the experimental results*. The other statements were not addressed by the intervention. To meet these LOs, only one curricular change was made, namely the replacement of the fourth lecture on tissue culture experimental details with a period of explicit instruction and practice toward the two LOs (Table 1).

The 50-min intervention included two active learning activities. To address LO1, each student submitted one draft results slide. The instructor grouped submissions into triads featuring figures/tables of variable quality. During class, students gave each other feedback in anticipation of revising the slides for their posters. The instructor also projected some sample slides for group discussion. Students did not use a rubric for this activity.

To address LO2, students were given figures and tables based on data derived from the tissue culture experiments. They analyzed the materials in small groups and wrote informative slide titles or stated the key results in short, specific phrases.

## Methods

### Overview of Research Questions

To evaluate the effect of the intervention of additional specialized instruction and practice in preparing figures and tables and writing about key results, data from the posters was systematically collected and analyzed. At the outset of the intervention, we hypothesized that peer-evaluation and self-evaluation would increase in accuracy relative to the instructor’s evaluation, and that overall student performance would increase. We posed three research questions (RQs) that compared the standard and intervention conditions:

#### RQ1.

Did the ability to accurately peer-review improve as a result of the teaching intervention?

#### RQ2.

Did the ability to accurately self-evaluate improve as a result of the teaching intervention?

#### RQ3.

Did student performance increase as a result of the teaching intervention?

### Data Collection

BIOE 342 posters were collected for this study over a six-year period, from 2007 to 2012. The data for each student’s poster included the following items: three peer-evaluations of the draft, one self-evaluation of the draft, one instructor evaluation of the draft, one instructor evaluation of the final. Peer- and self-evaluations, consisting of 14 statements, were gathered through CPR (Table 2). The instructor manually graded the draft and final posters using the same 14 statements. If the data for a poster was incomplete (e.g., missing a peer-evaluation), the poster was removed from the data set. Standard data was collected from 2007 to 2009 and yielded a total of 105 posters. Intervention data was collected from 2010 to 2012 and yielded 103 entries. All data was collected and used in compliance with university IRB requirements.

### Data Analysis for Statements 1–13 (Alphabetic, Categorical Data) for RQ1 and RQ2

For Statements 1–13, the responses were alphabetic, categorical data (e.g., A/B/C or Yes/No). Since each student submitted a unique poster based on his or her data, the posters could not be compared to a reference or standard poster. Thus, the instructor evaluation of the poster was established as the standard (since the instructor was the only constant factor across the multiple semesters of student submissions). Thus, the raw data for Statements 1–13 was reported as the *difference* between instructor and peer-evaluation or as the *difference* between the instructor and self-evaluation. To address RQ1, an analysis of peer-evaluation relative to instructor evaluation (PvI) was completed. To address RQ2, an analysis of self-evaluation relative to instructor evaluation (SvI) was completed.

#### Step 1: Create Bins as a Means to Compare PvI and SvI

Because the data was alphabetic and categorical (A/B/C or Yes/No), we characterized the difference between the instructor (the standard) and the peer (or self) scores using the terms “higher than,” “lower than,” and “agree.” Comparing the data was also complicated by the use of two different scales within CPR, a 3-point scale (A/B/C) and a 2-point scale (Yes/No). Since the divergence of the score given by the peer or self from the score given by the instructor could vary in magnitude, we adopted the terms “by 1” and “by 2” to represent the difference between the response and the standard. For statements using the 3-point scale (A/B/C) and 2-point scale (Yes/No), Table 3 captures the possible ratings and how they were categorized.

**Table 3:**
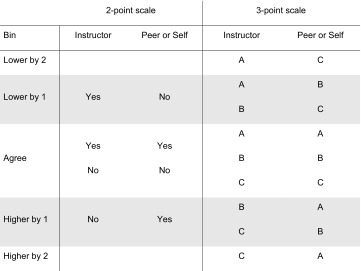
All possible 2-point and 3-point scale differences.

The bin is determined by the difference between the instructor and the peer or self. Given a Statement, the scores are defined: Yes as agree, No as disagree, A as high or strongly agree, B as moderate or neutral, C as low or strongly disagree. Peer or self scores are compared to the instructor’s scores. There are three bins on the 2-point scale, and five bins on the 3-point scale.

The bin was defined as the divergence of the peer (or self) relative to the instructor. Step 1 was executed four times for each poster, once for each of the three peer responses and once for the self-evaluation. To this end, rated data (A/B/C, Yes/No) from Statements 1–13 was transformed into binned data.

#### Step 2: Aggregate Bins across Each Statement

**Figure 1:**
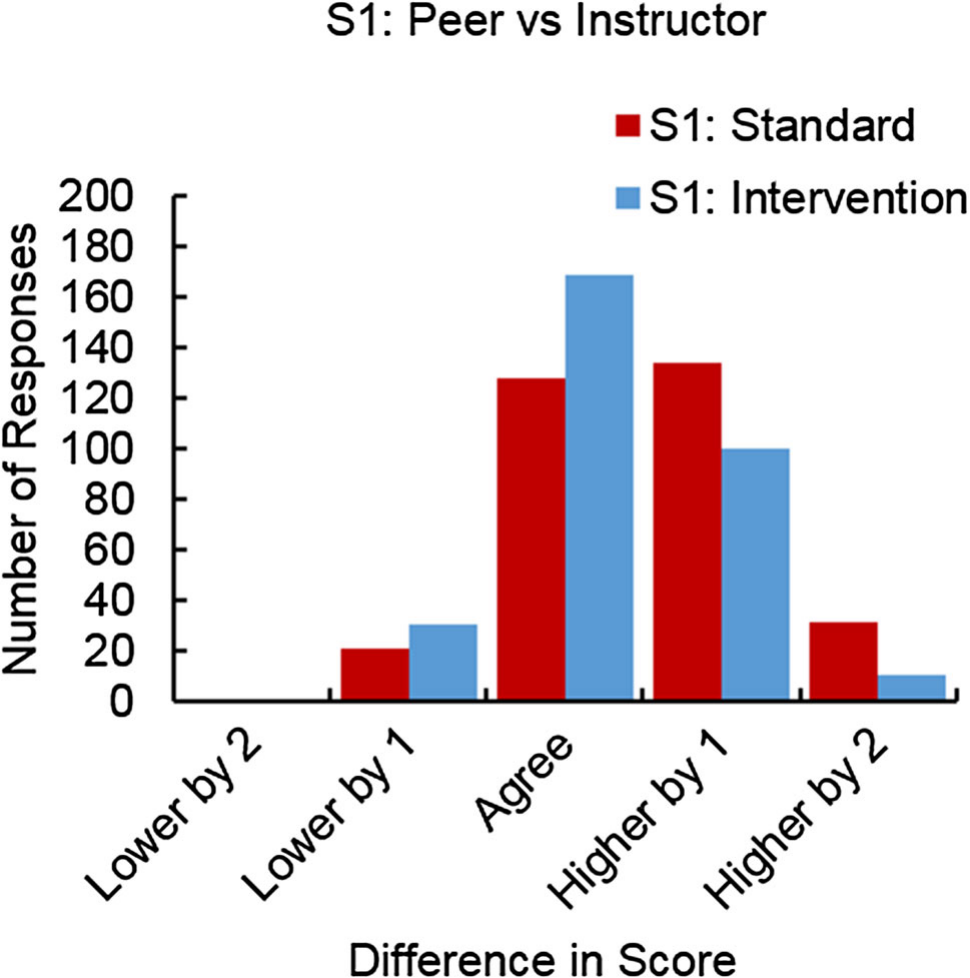
Comparison of the standard vs. intervention approaches for peer-evaluation of Statement 1 relative to the instructor. The number of responses is shown as a function of the difference in score. The bars show the number of times that the peer-evaluation was lower by 2, lower by 1, agrees with, higher by 1, or higher by 2, as compared to the instructor’s evaluation.

The second step in the data analysis was to sum the numbers within each bin to get a distribution for each statement. Data was summed within a treatment (i.e., standard or intervention) for the PvI or SvI. The output of the summations was a contingency table and a histogram for each statement that reported the number of responses as a function of bin (e.g., Fig. 1 represents PvI for Statement 1).

#### Step 3: Compare Standard vs. Intervention Conditions Using Chi Squared Test

Contingency tables presented the changes across treatment for peer vs. instructor (i.e., PvI) and for self vs. instructor (i.e., SvI). Contingency tables were evaluated with a *χ* ^2^ test to compare the standard and the intervention data under the null hypothesis that both sets came from the same population. The *χ* ^2^ test (alpha set to 0.05) was chosen for this alphabetic, categorical data because it uses the standard condition data to create expected values for each ordinal category in the intervention condition data.

The *P* value was calculated based on the *χ* ^2^ distribution and the degrees of freedom. A few of the contingency tables expressed random zeros. When this occurred, a small constant of 0.00001 was added to every cell in the contingency table.^3^

#### Step 4: Test for Convergence

A test for convergence was completed only if the *P* value was <0.05. The desired change was a convergence of responses to the “Agree” bin, as this showed an improvement in a student’s ability to match the instructor’s evaluation ratings. In order to show convergence after intervention, the “Agree” bin must have contained a higher number of responses, and the other bins must have shown a lower number of responses.

### Data Analysis for Statement 14 (Continuous, Numerical Data) for RQ1 and RQ2

Statement 14 evaluated the overall quality of the poster. Peer- and self-evaluation data was collected through CPR on a 1–10 scale. Overall poster quality grades were given by the instructor on a 100-point scale (range 60–96). These grades were linearly scaled down to a 1–10 scale using the formula: scaled score = (instructor grade %—60%)/4 + 1. For example, A (96%) = 10, A − (92%) = 9, …, D + (64%) = 2, D (60%) = 1. For Statement 14, the responses were assumed to be continuous.

For the PvI comparison, the mean of three peer-evaluation scores for Statement 14 was calculated and graphed against the instructor score in scatterplots. Because data was continuous, averages (rather than differences) were used to present values. For both the standard and intervention conditions, linear regression equations and *r* values were computed. Convergence to instructor scores is shown by a movement towards a slope of 1 in the regression equation and an increase in the *r* value (i.e., closer to 1) from the standard to the intervention condition. For the SvI comparison, the analysis of Statement 14 was almost identical to the PvI comparison, except no averages were taken, since there was only one self-evaluation per poster.

### Data Analysis for Statements 1–13 for Instructor Scores for RQ3

To address RQ3, an analysis of the instructor’s scores before and after the intervention was conducted. Specifically, this analysis examined raw instructor scores for Statements 1–13 during the standard and intervention conditions. The four-step process outlined in the “Data Analysis for Statements 1–13 (Alphabetic, Categorical Data) for RQ1 and RQ2” section was used.

To check the direction of this shift, the “mean” of the scores was calculated for the standard and intervention conditions. The A/B/C responses were coded as 3/2/1, respectively and the Y/N responses to 2/1, respectively. Averages of these scores for each statement were then calculated and compared between conditions. A higher average in the intervention condition confirmed that the score for this statement improved following the intervention.

## Results

Throughout the Results section, Statement 1, “Key results are presented clearly in graphs and tables,” is used as the exemplar figure for histogram data. Table 4 summarizes the data for all RQs as they relate to LO1 and LO2 and Statements 1–13.

**Table 4:**
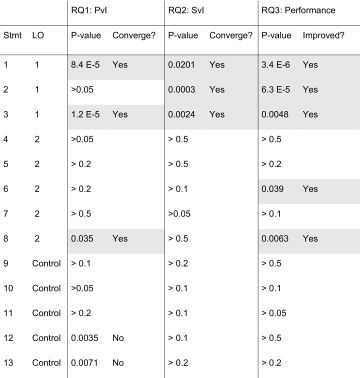
Results of statistical analysis for statements (Stmt) 1–13 organized by learning outcome (LO).

Results show *P* value comparison of standard vs. intervention approaches for research question 1 (RQ1) (peer-evaluation of posters relative to the instructor), RQ2 (self-evaluation of posters relative to the instructor), and RQ3 (student performance, as assessed by the instructor). Statistical significance, reported as a *P* value, was evaluated with a *χ* ^2^ test. Convergence (i.e., student resembled instructor) and improved performance (i.e., performance shifted toward a higher score) was tested when *P* < 0.05. Convergence or improvement is marked as Yes or No, with blank as N/A (i.e., *P* value not significant). For easy visual reference, blocks are shaded when there is a statistically significant difference and convergence (RQ1 and RQ2) or improvement in performance (RQ3).

### Effects of Teaching Intervention on Students’ Ability to Peer Review (RQ1)

Figure 1 shows how peer-evaluation was lower than, in agreement with, or higher than the instructor’s evaluation for Statement 1 PvI. Using the standard teaching method, students overrated peers in 165 of 315 times (52%). In 128 of 315 times (41%), the peer and instructor assigned the same score. In 22 of 315 times (7%), students underrated their peers relative to the instructor.

After the intervention, there was a shift in the distribution of peer-evaluation as compared to instructor evaluation. Overall, the percentage of students who overrated their peers as compared to the instructor decreased from 52% to 36% (110 of 309 times). The number and percentages of students who agreed with the instructor increased from 40% to 55% (169 of 309 times). After the intervention, 10% of the peers’ ratings for Statement 1 were more critical than the instructor’s.

As noted in Table 4, the *P* value for Statement 1 was 0.000084. This difference converged, meaning there was a shift toward the center. This indicates a statistically significant change in distribution towards greater agreement with the instructor after the intervention.

Contingency tables and related data analysis were completed for Statements 1–13. As shown in Table 4 in the RQ1: Peer vs. Instructor column, statistically significant changes were seen for LO1 (Statements 1, 3), LO2 (Statement 8) and control (Statements 12–13). For Statements 1, 3, and 8, statistically significant differences were shown to converge, meaning that evaluation of their peers’ work aligned more closely with the instructor’s evaluation after the intervention. For Statements 12–13 (controls), convergence was not seen.

**Figure 2:**
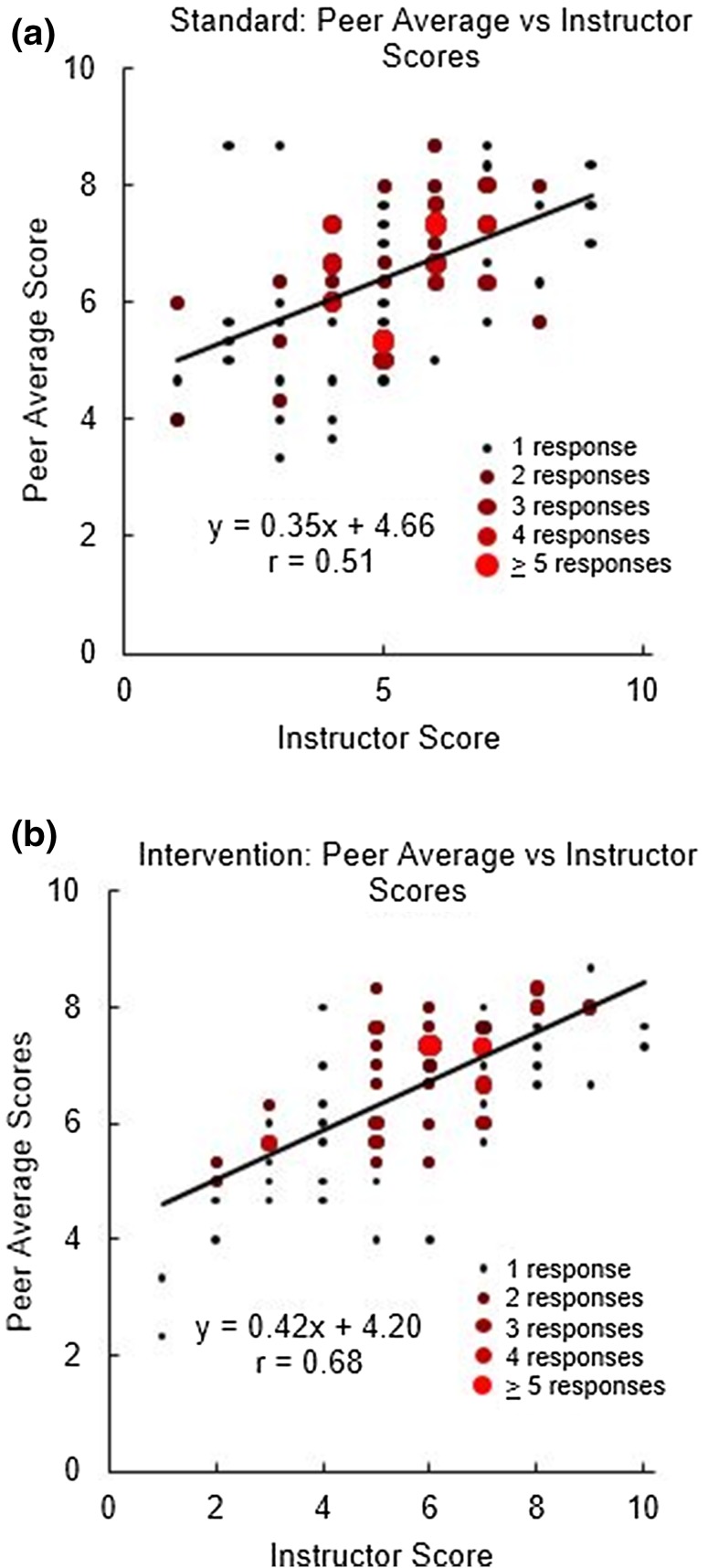
Scatterplots of Statement 14, the overall score of the poster (on a 1–10 scale), for the standard (a) and the intervention (b). For each poster, three peer scores are averaged to one value, which is plotted against the instructor score. The size and color of the dot represent the number of responses at that location.

LO Overall (Statement 14) required a holistic score of the overall quality of the poster, “How would you rate this text/poster?” Figure 2a is a scatterplot of the average peer score vs. the instructor score for the standard condition. For the standard condition, there was a weak correlation (*r* = 0.51) between the average peer score and the instructor score. As shown in Fig. 2b, the correlation improved to *r* = 0.68 for the intervention condition. The shift toward better correlations shows students align better with instructor on the holistic grade of the poster. Overall, peers generally gave higher scores than the instructor, particularly for the posters that the instructor scored the lowest.

### Effects of Teaching Intervention on Students’ Ability to Self-Evaluate (RQ2)

Contingency tables and histograms to compare students’ ability to evaluate their own work against the standard of the instructor were created for Statements 1–13 using a similar method to RQ1. The data analysis from these graphs is summarized in Table 4 under the RQ2: Self vs. Instructor column. Statistically significant changes after intervention were seen in all statements for LO1 (Statements 1–3). All of these changes were shown to converge, which means for these three statements, a student’s evaluation of his or her own work more closely matched the instructor’s evaluation after the intervention. There was no difference or convergence for statements related to LO2 or control.

To evaluate Statement 14, we generated plots similar to Fig. 2 for self-evaluation score vs. instructor score (data not shown). For the standard condition, there was no correlation between self- and instructor evaluation (*r* = 0.18). After the intervention, the *r* value rose to 0.37, suggesting a weak correlation. Again, students generally gave higher scores to their own work than the instructor did.

### Effects of Teaching Intervention on Students’ Ability to Present Key Results (RQ3)

**Figure 3:**
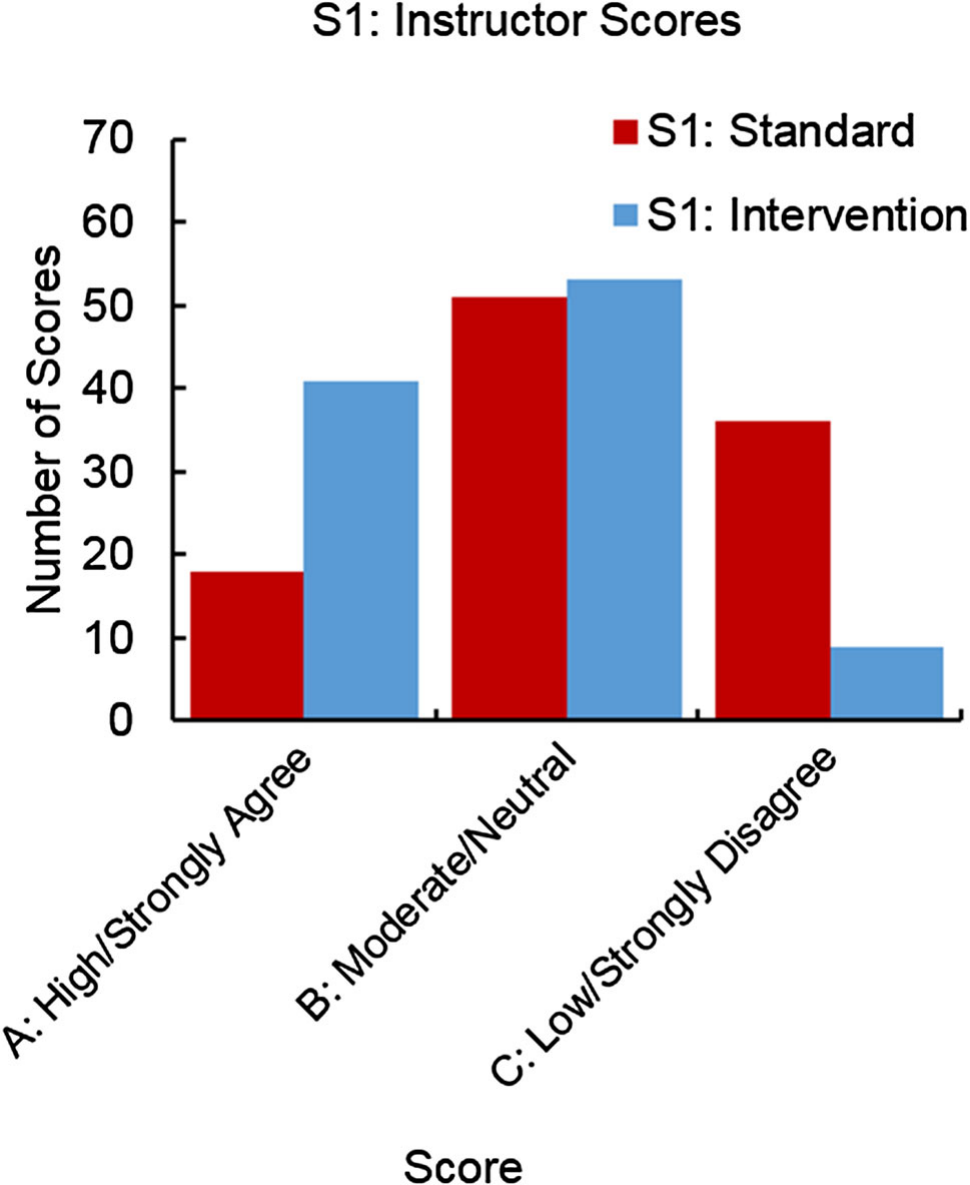
Instructor scores before and after intervention for Statement 1. A score of A indicates high agreement with the statement; a score of B indicates moderate agreement, and a score of C indicates low agreement.

Figure 3 presents CPR scores given by the instructor to the students’ posters on Statement 1 before and after the intervention. During the standard administration, 17% of the students scored an A (high/strongly agree), 49% of the students scored a B (medium/neutral), and 34% scored a C (low/strongly disagree). After the intervention, the scores shifted dramatically. Forty percent scored an A, 51% scored a B, and only 9% scored a C. Thus, the instructor’s ratings indicate that students substantially improved in their ability to create graphs and tables that clearly present the key results.

As shown in Table 4 under RQ3: Student Performance, statistically significant changes were evident in LO1 (Statements 1–3) and LO2 (Statements 6, 8). For all five statements, student performance improved. There was no difference or shift in direction for statements related to the control.

## Discussion

### Using CPR™ to Assess Pedagogical Interventions

We successfully leveraged CPR’s functionality in a novel way to identify and address gaps in student learning related to the critical analysis of experimental data and technical communication. In our previous work,^22^,^26^ we analyzed the peer review and self-evaluation data collected in CPR. This analysis revealed good alignment for PvI and SvI ratings on statements requiring comprehension and application of knowledge, as well as on the statement asking for an overall rating of a poster’s quality.^26^ In order to improve the quality of the course and student performance, we designed educational interventions to target poor alignment related to higher-order thinking tasks, specifically in data presentation and interpretation.

Importantly, we used CPR in the revised course design to measure changes in students’ performance. We found that after guided practice on figures and tables, students showed better alignment with the instructor on some statements and that their skills improved in particular areas. Ultimately, CPR provided a powerful platform that enabled us to hone in on specific weaknesses in students’ visual communication skills and to determine whether a new pedagogical approach yielded better outcomes. With its automated, online system, CPR can be a helpful tool for engineering educators as they seek to deliver customized instruction to their students.

### Mixed Intervention Improved Students’ Visual Communication Skills

In a laboratory setting, a combination of interventions can help students develop their “scientific literacy,” which includes the student’s ability to collect data, analyze results, and understand cause and effect in an experiment.^20^ In a pedagogical intervention similar to ours, Carter showed that explicit instruction and practice using LabWrite helped students with many aspects of lab reports, including representing and interpreting data.^7^ In another example, Taylor and Builford adopted a critique approach in which bioengineering capstone design students provided formative feedback to their peers on figures and tables in reports.^25^ Like us, they taught a workshop on the principles of visual design and had students apply them to critique the work of their peers. This approach resulted in more informative figure captions, although it is not clear whether the improvements extended to data interpretation. They also observed improvements in clarity and readability, but not statistically significant changes.

The pedagogical intervention described above has important features that promote student learning. The “lecture” period that addressed the LOs was designed based on active learning strategies, including cooperative learning, which have been shown to be successful in promoting student learning and mastery of material.^11^,^19^ In a meta-analysis of 117 studies and over 20,000 participants, Abrami asserts that a “mixed” intervention of (1) guided instruction on critical thinking and (2) immersion of critical thinking in the discipline has more impact on student performance than either intervention given independently.^2^ During the intervention, we taught using a “mixed” approach by combining authentic work in a tissue culture lab, direct instruction on visual communication, and practice on creating and critiquing materials.

In the intervention, we gave explicit instruction on visual communication skills to students by guiding them to practice data visualization and interpretation in small peer groups. After the intervention, we saw significant improvements in peer- and self-evaluation and performance in LO1 (Development of Graphs and Tables) for the majority of the categories (specifically, eight of nine comparisons). We postulate that these significant improvements are related to the nature of the intervention assignment. Students created their own graphs or tables for this activity and then had the opportunity to critique their work in small groups. We speculate that this level of personal investment in the graph, the dialogue in which they engaged about their work, and the knowledge they could make improvements to it for their graded poster draft contributed to such substantial growth around this LO.

On the other hand, the intervention did not result in significant improvements in peer- and self-evaluation and performance for LO2 (Interpreting and Summarizing Results). In fact, improvements were seen in only 3 of 15 comparisons. Overall, students did not become noticeably better or worse at interpreting or summarizing experimental results. One possible reason for this may have been the nature of the intervention, where students wrote key results and titles based on tables and graphs developed by the instructor. We postulate that students’ level of engagement in this activity may have diminished because they were not reviewing and revising their own work. Another possible reason is that the time devoted to this activity (20–25 min) was insufficient for students to make substantial progress on this difficult task. Furthermore, students may have not have gained additional proficiency in interpreting experimental results because one 50-min lecture covering technical content on tissue culture experiments was eliminated. On the other hand, the reduction in technical content appeared to have no detrimental effect. To improve the outcomes related to LO2, we think it would be beneficial to spend more time on interpreting and summarizing results and to use the students’ own work instead of instructor-generated examples.

As an important control, we considered impacts to Statements 9–13 that were not linked to either LO (Table 4). Since the majority (13 of 15 comparisons) did not show significant improvement, we can conclude that an intervention that targets one aspect of student learning does not positively or negatively affect student performance in other areas of the assignment. That said, two of 15 comparisons, namely Statements 12 and 13 for RQ1 (PvI), did return significant results, but without convergence. This means that as compared to the instructor, the peer evaluations got worse after the intervention for these two statements. We cannot readily explain this. However, we did not see any significant difference for Statements 12 and 13 for RQ2 or RQ3. If we had seen a similar decline for RQ3, which is related to performance, we would have been concerned; however, this was not the case.

### Correlation between Student and Instructor Ratings Improved

Moving forward, we found that student and faculty ratings were more closely aligned after the intervention. The overall grades given by peers and instructors (Fig. 2a–b) presented several interesting trends. In Fig. 2, the slope is less than 1 (where 1 would be perfect agreement) and the intercept is between 4 and 5, suggesting that overall grades from peers are higher than the instructor’s. This result was consistent with our previous work that showed that students overestimate the quality of their peers’ work.^26^ After the intervention, the correlation coefficient increased from *r* = 0.51 (Fig. 2a, pre-intervention) to *r* = 0.68 (Fig. 2b, post-intervention), demonstrating a modest improvement. The scatterplot and fits of the overall grades of SvI are not shown, but we observed similar trends, with *r* = 0.18 for pre-intervention and *r* = 0.37 for post-intervention. As shown in our previous work, the correlation coefficients were lower for self-evaluation than for peer-evaluation.^26^

The post-intervention results are also consistent with Falchikov and Goldfinch’s impressive meta-analysis of 48 peer-reviewed studies, from which they concluded that a strong correlation (mean *r* = 0.69) exists between instructors and peers in determining global ratings.^10^ Based on their work^10^ and ours, we suggest factors that can improve student/instructor alignment and, ultimately, student performance: increased guided instruction time on challenging tasks, a collaborative learning environment, and increased practice by students where they apply key principles in the domain of interest.
